# Orbital-specific Tunability of Many-Body Effects in Bilayer Graphene by Gate Bias and Metal Contact

**DOI:** 10.1038/srep03713

**Published:** 2014-01-16

**Authors:** Hirokazu Fukidome, Masato Kotsugi, Kosuke Nagashio, Ryo Sato, Takuo Ohkochi, Takashi Itoh, Akira Toriumi, Maki Suemitsu, Toyohiko Kinoshita

**Affiliations:** 1 Research Institute of Electrical Communication, Tohoku University, 2-1-1 Katahira, Aobaku-ku, Sendai, Miyagi 980-8577, Japan; 2 JASRI/SPring-8, 1-1-1 Kouto, Sayo, Hyogo 679-5198, Japan; 3 Department of Materials Engineering, Graduate School of Engineering, University of Tokyo, 7-3-1 Hongo, Tokyo 113-8656, Japan; 4 Frontier Research Institute for Interdisciplinary Sciences, Tohoku University, 6-3 Aoba, Aoba-ku, Sendai, Miyagi 980-7578, Japan

## Abstract

Graphene, a 2D crystal bonded by π and σ orbitals, possesses excellent electronic properties that are promising for next-generation optoelectronic device applications. For these a precise understanding of quasiparticle behaviour near the Dirac point (DP) is indispensable because the vanishing density of states (DOS) near the DP enhances many-body effects, such as excitonic effects and the Anderson orthogonality catastrophe (AOC) which occur through the interactions of many conduction electrons with holes. These effects renormalize band dispersion and DOS, and therefore affect device performance. For this reason, we have studied the impact of the excitonic effects and the AOC on graphene device performance by using X-ray absorption spectromicroscopy on an actual graphene transistor in operation. Our work shows that the excitonic effect and the AOC are tunable by gate bias or metal contacts, both of which alter the Fermi energy, and are orbital-specific.

Graphene, a honeycomb network consisting of carbon atoms bonded by π- and σ-orbitals, is a model two-dimensional (2D) compound in physics^1^,^2^,^3^, electronics^4^,^5^,^6^ and chemistry^7^ due to its exceptional electronic properties, such as linear band dispersion^1^,^2^,^3^,^4^ and very high carrier mobilities^2^. A promising application of graphene is in optoelectronic devices^8^, such as mode-locked lasers^9^ and current-injection graphene terahertz lasers^10^, and this use requires an understanding of the behaviour of quasiparticles near the Dirac point (DP) in graphene^11^.

Many-body effects, such as electron-electron interactions, are prominent in graphene owing to a limited density of states (DOS) near the DP^3^, resulting in the enhancement of Coulomb interactions and reduced screening^12^. These effects significantly alter the behaviour of quasiparticles in graphene^12^, as manifested by the renormalization of the Fermi velocity^13^ and band structure^14^, and by excitonic effects in optical response^15^. Similarly, the behaviours of quasiparticles in bilayer graphene are altered also by many-body effects, such as excitonic effects^16^ and renormalizations of the Fermi velocity^17^ and phonon self-energy^18^. The many-body effects are tunable by varying the Fermi energy E_F_^12^; they are enhanced near the DP, while they are weakened away from the DP. The position of the Fermi level varies with the key parameters for optoelectronic device operation, such as gate bias and metal contact properties^19^. The many-body effects are therefore likely to affect optoelectronic device performance.

An orbital-specific investigation is vital for the study of the many-body effects in graphene because they critically depend on the molecular orbitals involved. X-ray absorption spectroscopy (XAS) is a suitable tool for this as it can provide an element- and orbital-specific probe of electronic structure affected by dynamical many-body effects during the absorption processes, including excitonic effects^20^,^21^ and the Anderson orthogonality catastrophe (AOC)^22^,^23^. Although XAS has been applied to graphene^24^,^25^,^26^,^27^, XAS studies of a graphene device in operation have not yet been performed.

Here we present the results of microscopic XAS (μ-XAS) studies on an actual graphene transistor in operation. We demonstrate the orbital-specificity and tunability of excitonic effects and the onset of the AOC using gate bias or metal contacts, both of which alter E_F_.

## Results

### Effect of applying a gate bias

**Figure 1:**
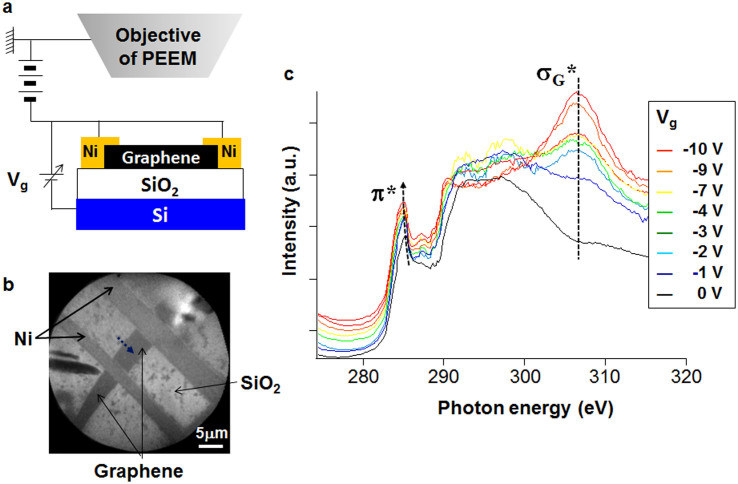
Variation of μ-XAS spectra of graphene with gate bias (V_g_). (a) Schematic drawing of the μ-XAS observation system. (b) A typical PEEM image of the graphene device. The blue dotted arrow indicates the μ-XAS measurement position. (c) The μ-XAS spectra of the graphene at different V_g_.

In the graphene device used in this work, bilayer graphene (see Supplementary Fig. S1) and Ni thin films were used as the channel and metal electrodes, respectively (see Fig. 1). The gate bias (V_g_) was applied using a SiO_2_ thin film as a capacitor and a Si substrate as a backgate. Under our experimental conditions, a negative gate bias means positive charging of graphene and therefore hole doping. The μ-XAS spectra were acquired by using a photoemission electron microscope (PEEM) whose lateral resolution was 22 nm in the Auger electron yield mode^28^.

**Figure 2:**
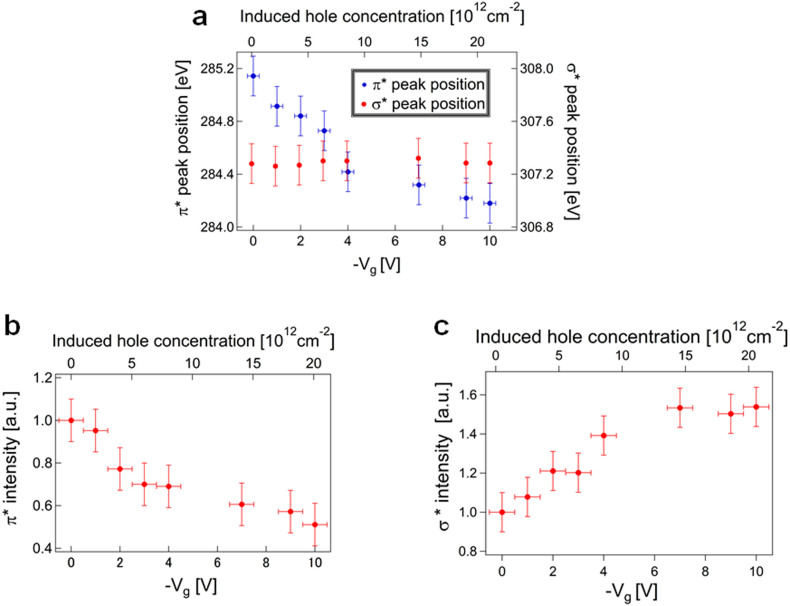
Change of π* and σ_G_* with gate bias. (a) Changes of π* and σ_G_* peak positions with varying gate bias. (b) Reduction of π* peak intensity with increasing gate bias. (c) Enhancement of the σ_G_* peak intensity with increasing gate bias.

Figure 1c shows μ-XAS observations taken at the centre of the graphene for different values of gate bias. The μ-XAS spectra are divided into two parts, consisting of π* (~285 eV) and σ* (290–315 eV) peaks^20^,^21^,^22^,^23^,^24^,^25^,^26^,^27^. The π* peak is due to the excitation from the C1s core level into unoccupied states of the π orbital, that is, the conduction band in graphene. The σ* peaks are due to the excitations from the C1s core level to unoccupied σ orbitals. The observed line shapes of the μ-XAS spectra are similar to those of the spectra of bilayer graphene reported previously^25^, thus confirming that our μ-XAS measurements using the PEEM probed the electronic structure of the graphene in the device. The π* (~285 eV) and σ_G_* (~307 eV)^29^ peaks are strongly affected by changes in gate bias, as shown in Figure 1c. Although the π* peak position and intensity are both altered, the σ_G_* peak shows a change in only peak intensity. The π* peak position is redshifted by increasing the gate bias, as shown in Figure 2a. In a non interacting system, the π* peak is determined by the ground-state π* orbital without a core hole^20^,^21^. The π* peak position should then be at 287.5 eV^20^, which disagrees with our experimental value (~285 eV). This discrepancy is due to the creation of an exciton, a pair consisting of many electrons and a core hole produced during X-ray absorption^20^,^21^,^27^. According to the Mahan-Nozières-deDominicis theory^22^,^23^ which is in principle applicable to the entire spectrum^21^,^30^, not just near the Fermi edge threshold, by assuming the creation of the core hole as fast enough process but takes into account the real dynamics of the response of the electronic subsystem to the core hole creation, a redistribution of the spectral density of the π* peak occurs in graphene^21^, but no singularity at the Fermi edge threshold can be seen because of the limited DOS near the DP^21^, in contrast to the case of typical metals, such as Mg, in which the singularity is seen owing to the appreciable amount of DOS near E_F_^22^,^23^. The excitonic effect renormalizes the peak position (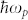) in the non-interacting system to be 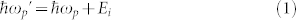
where *E_i_* is the self-energy of the core hole, which lowers the peak position^31^. This modification accounts for the increased binding of the core hole due to the polarization of electrons around it^31^. This polarization varies with E_F_^32^, such that the peak position is altered by E_F_. The redshift induced by increasing the gate bias is in fact corroborated by a recent theoretical calculation of X-ray absorption spectra of graphene into which positive charges are doped^33^.

The π* peak intensity is reduced as the gate bias is increased (Fig. 2b). This change cannot be explained by the excitonic effect alone, which enhances the peak intensity^22^. According to the Nozières-deDominicis theory^22^,^23^, the XAS intensity *A*(*ω*) follows a power law^23^,^31^: 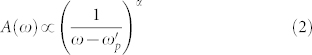
where the exponent *α* is governed by the AOC as well as the excitonic effect^22^,^23^,^31^. The former, which reduces the intensity, is rationalized as follows^23^,^31^. The initial state of the X-ray absorption processes consists of the core electron state and many conduction-electron states. In the final state, an electron is excited from the core electron state into one of the many conduction-electron states. The local potential induced by the core hole slightly alters the wave functions of all the conduction electrons, such that the initial and final states are orthogonal to each other. This means that the matrix element connecting the initial and final states tends to zero as the number of states approaches infinity. The contribution of the AOC to the exponent (*α_A_*) is thus given in terms of the DOS at E_F_ as^31^

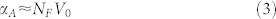
where *N_F_* and *V*_0_ are the DOS at the Fermi level and the screened Coulomb interaction, respectively^31^. Notably, the AOC is suppressed when E_F_ is situated near the DP because of the vanishing of *N_F_*^34^, while it is recovered by moving E_F_ away from the DP owing to the increase of *N_F_*^34^. The increased contribution of the AOC to the power law exponent as E_F_ moves away from the DP thus accounts for the reduction of the π* peak intensity.

To scrutinize the orbital-specificity of the many-body effects, the change of the σ_G_* peak by the gate bias application is examined. As shown in Figure 1c and Figure 2a, the σ_G_* peak position is unchanged when varying the gate bias. According to a previous theoretical work^20^,^29^, the π* excitation has been identified as a localized Frenkel-like exciton consisting of core hole and electron^20^, and hence experiences a significant peak shift. On the other hand, the higher-lying excitations such as σ_G_* peak are to delocalized conduction band states^29^, and are not affected by the excitonic effect not so much as the π* excitation^29^. Hence, the invariance of the σ_G_* peak position is related with the absence of the excitonic effect for the σ_G_* orbital. It is therefore the ground-state unoccupied DOS (UDOS) without the influence of the core hole which is reflected in the line shape of the σ_G_* peak, in contrast to the π* peak whose shape is determined by the excited-state UDOS with the influence of the core hole^21^.

Figure 2c shows the gate-bias dependence of the σ_G_* peak intensity. The intensity is enhanced by increasing the gate bias, in contrast to the depression of the π* peak intensity (Fig. 2b). Because of the absence of the excitonic effect, the intensity enhancement must be attributed to a perturbation of the ground-state UDOS responsible for the absorption process in the σ_G_* peak. One candidate for this perturbation is the polarization of UDOS, which was suggested in a previous XAS study of an organic thin film transistor^35^. When polarization is induced, UDOS can undergo a change depending on the applied electric field^35^. As a result the polarization modifies the overlap of the core level and UDOS which determines the intensities of some of the components in the XAS spectra. In our work, the surface-normal component of the σ_G_* peak is larger than the surface-parallel component (see Supplementary Fig. S2). This surface-normal component of the σ_G_* orbital is perturbed by the surface-normal electric field produced by the gate-bias application, resulting in the enhancement of the σ_G_* peak. In contrast, the π* peak is not significantly affected by this perturbation^35^. This could be due to the fact that the π* peak is determined by the excited-state DOS with the core hole, while the σ_G_* peak is determined by the ground-state UDOS which is perturbed by the electric field. The above results for peak position and intensity indicate that, unlike the π* orbital, the σ_G_* orbital is not strongly influenced by the many-body effects.

### Effect of contact with metal

**Figure 3:**
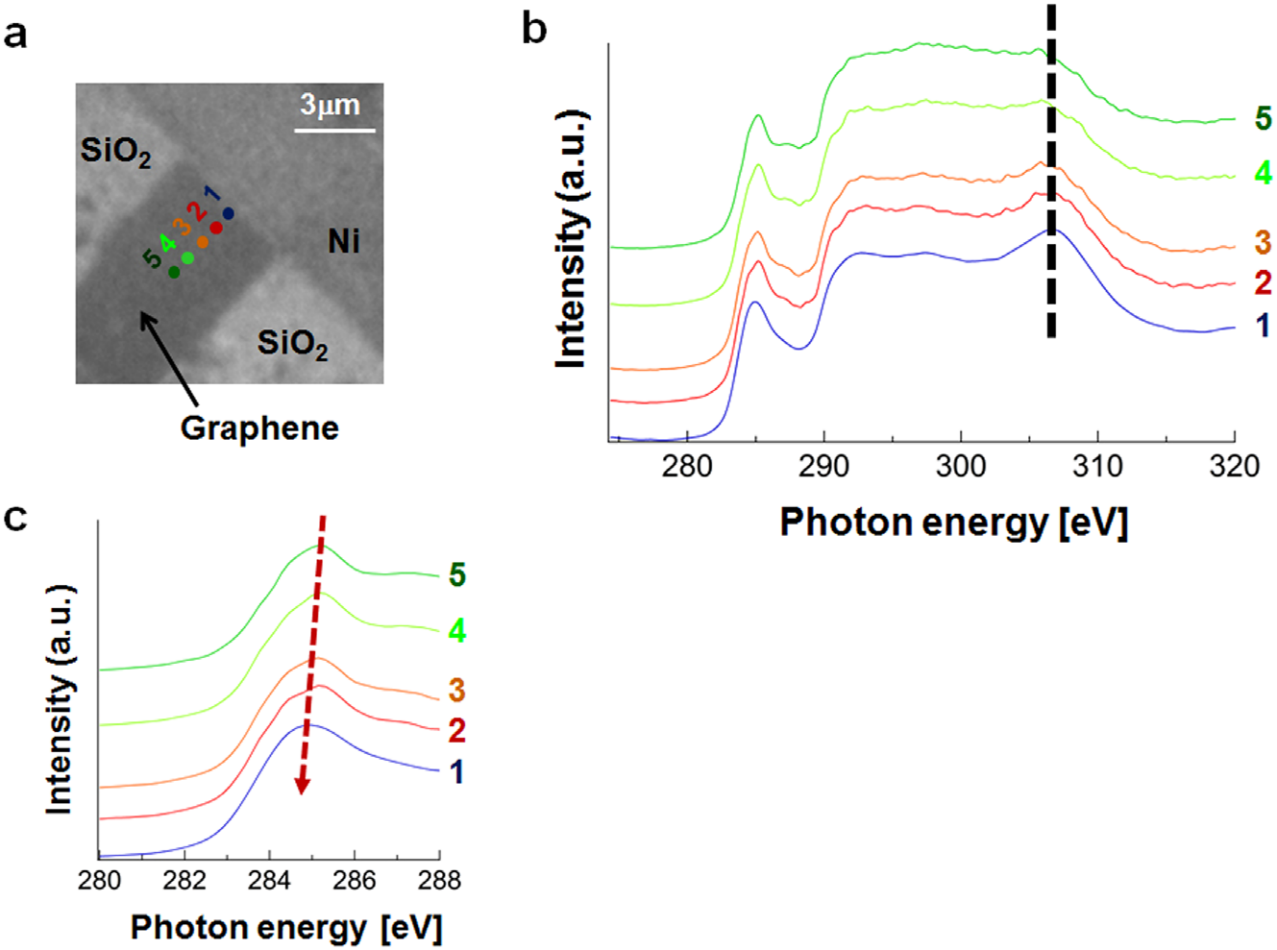
μ-XAS spectra of graphene near the metal contact. (a) Typical PEEM image of the graphene to show the region from which the μ-XAS spectra were taken. (b) μ-XAS spectra near the metal contact at a gate bias of 0 V. (c) Detailed view of the spectra near the π* peak.

Carrier doping is also induced through contact with metal^5^,^19^; for example, contact with a Ni electrode induces hole doping in graphene^19^. Carrier doping leads to the formation of appreciably wide charge transfer regions due to the vanishing DOS near the DP, as suggested by previous reports on the performance of graphene transistors^19^. As demonstrated above, the many-body effects on the π* peak are modulated by carrier doping through the application of a gate bias. Accordingly, the many-body effects on the π* peak are also expected to be modified near the interface between graphene and a Ni electrode. Figure 3 shows that the π* peak position near the interface exhibits a redshift due to hole doping, which is similar to the effect of varying the gate bias.

**Figure 4:**
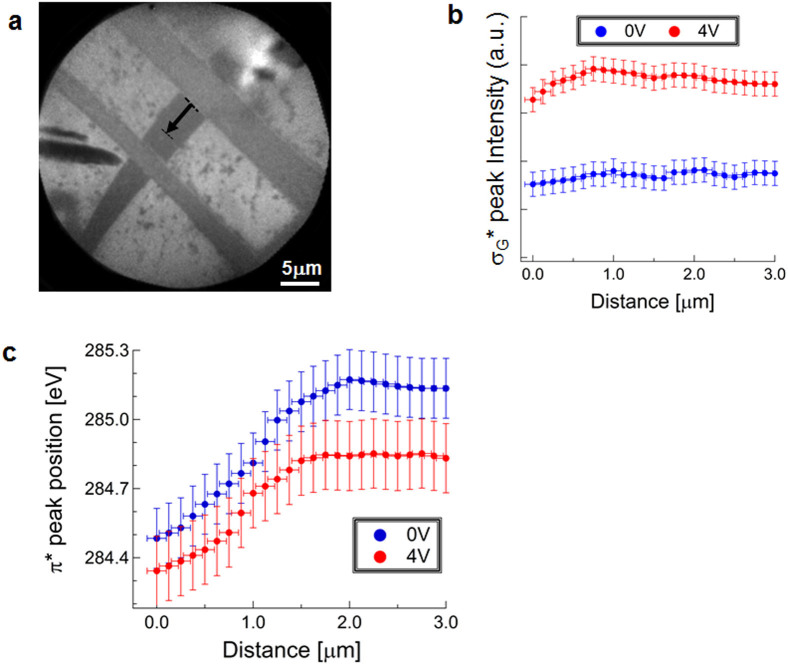
Change of π* and σ_G_* near the contact with metal with different gate biases. (a) A PEEM image to show where the line profiles were taken. (b) The variation of the σ_G_* peak intensity near the contact. (c) The variation of π* peak position near the contact.

The orbital-specificity of the many-body effects due to the metal contact can be seen by considering the σ_G_* peak. Figure 3b shows that the σ_G_* peak position is not strongly affected by the presence of the graphene-Ni contact. This invariance indicates the absence of the excitonic effect just as it did when we varied the gate bias. Figure 4b shows the variation of the σ_G_* peak intensity near the graphene-Ni interface for different gate biases. Although the intensity is enhanced by applying a gate bias, the variation near the contact is less strong. The gate bias produces a stronger electric field across the SiO_2_ thin film (90 nm thick), in the order of 10 V/μm. On the other hand, our previous scanning photoelectron microscopy on graphene/SiO_2_/Si in contact with Ni electrodes shows that the electric field near the contact is in the order of 0.1 V/μm^36^. This weaker electric field is ascribable to the reason for the smaller variation of the σ_G_* intensity. We have thus shown here that the contact of graphene with a metal produces an orbital-specific modulation of the many-body effects in a way similar to the application of a gate bias.

## Discussion

An important observation is that the π* peak position at the graphene-Ni interface is not pinned for different gate biases (see Fig. 4c). This behaviour is related to the so-called Fermi-level (de)pinning. The π* peak position in the μ-XAS spectrum of graphene is determined by the excitonic effect which is affected by E_F_, as explained above. The variation of the π* peak position at the interface therefore indicates that the value of E_F_ at the interface varies with gate bias. This demonstrates that Fermi-level pinning, which has been controversial^37^, does not occur under our conditions for fabricating the metal contact.

Also worth noting is how the redshift of the π* peak near the interface, that is, in the charge transfer region, varies with gate bias. As can be seen in Figure 4c, the region where the redshift of the π* peak varies near the contact is narrowed by the gate bias application. This narrowing of the charge transfer region is corroborated by considering that the screening length (λ), given by λ = [4πN_F_]^−1/2^ 19, decreases as *N_F_* is increased by moving E_F_ away from the DP.

In conclusion, we have demonstrated that graphene possesses orbital-specific many-body effects, which vary with E_F_ through application of a gate bias or contact with a metal, by using μ-XAS on an actual graphene transistor in operation. Since the many-body effects are involved dynamically in photoabsorption processes, our work is important for an understanding device characteristics, such as output characteristics of lasers and the energy distribution of emitted photons which is related to the (renormalized) DOS and band structure.

## Methods

In this work, the device consisted of bilayer graphene, a SiO_2_ thin film (90 nm thick) used as a capacitor, a p-Si(100) substrate used as the backgate and Ni thin film electrodes in contact with the graphene. The bilayer graphene was exfoliated from Kish graphite and transferred onto the SiO_2_ thin film^19^. The Ni electrodes were thermally evaporated onto the resist-patterned graphene in a chamber with a background pressure of 10^−5^ Pa and were subjected to a lift-off process in warm acetone. The gate bias was applied between the graphene and the Si backgate. The graphene in this work was positively charged so it could be doped with holes by applying a negative gate bias.

The μ-XAS spectra were measured by the Auger electron yield method using a PEEM (LEEM III, Elmitec GmbH) installed at BL17SU at SPring-8^38^. The μ-XAS spectra were corrected by using mirror current monitoring the incident x-ray intensity and the spectra of Cu(100) substrates taken just after the μ-XAS measurement of the graphene in the same experimental condition. The energy resolution of the incident X-rays (E/ΔE) is better than 4,000^39^. The lateral resolution of the μ-XAS is down to 22 nm. For the μ-XAS measurement, the incident X-rays were elliptically polarized unless otherwise noted. The μ-XAS spectra are normalized by the intensity of the pre-edge region (around 278 eV).

## Author Contributions

H.F. assisted with sample preparation, performed μ-XAS experiments, participated in designing the study, and participated in writing of the manuscript; M.K. performed μ-XAS experiments, participated in designing the study, and participated in writing of the manuscript; K.N. undertook sample preparation, participated in designing the study, and participated in writing of the manuscript; R.S. performed μ-XAS experiments; T.O. performed μ-XAS experiments; T.I. participated in designing the study, and participated in writing of the manuscript; A.T. oversaw sample preparation, participated in writing the manuscript; M.S. assisted with writing the manuscript; T.K. participated in writing the manuscript and discussed about the content of the manuscript.

## Supplementary Material

Supplementary Information Supplementary information
