# Supplementary material for: Orbital-specific Tunability of Many-Body Effects in Bilayer Graphene by Gate Bias and Metal Contact
Source: Sci Rep. 2014 Jan 16;4:3713. doi: 10.1038/srep03713 (PMC3893642; doi:10.1038/srep03713)
Supplement: Supplementary Information [file srep03713-s1.pdf]

SUPPLEMENTARY INFORMATION

Orbital-specific Tunability of Many-Body Effects in Bilayer Graphene by Gate Bias and Metal Contact

Hirokazu Fukidome[1*], Masato Kotsugi[2], Kosuke Nagashio[3], Ryo Sato[1], Takuo Ohkochi[2], Takashi Itoh[4], Akira Toriumi[3], Maki Suemitsu[1], Toyohiko Kinoshita[2]

[1]Research Institute of Electrical Communication, Tohoku University, 2-1-1 Katahira, Aobaku-ku, Sendai, Miyagi 980-8577, Japan

[2]JASRI/SPring-8, 1-1-1 Kohto, Sayo, Hyogo 679-5198, Japan

[3]Department of Materials Engineering, Graduate School of Engineering, University of Tokyo, 7-3-1 Hongo, Tokyo 113-8656, Japan

[4]Center for Interdisciplinary Research, Tohoku University, 6-3 Aoba, Aoba-ku, Sendai, Miyagi 980-7578, Japan

*Corresponding author: fukidome@riec.tohoku.ac.jp

S1. Characterization of graphene device

Figure S1a shows an optical micrograph of the graphene device, which consists of the graphene, a SiO_2 (90 nm thick) insulator, a Si(100) substrate used as the backgate and Ni thin films used as the metal electrodes. In the optical micrograph, the graphene is visible due to the interference effect[1]. The bilayer graphene in the device was characterized by Raman microspectroscopy, as shown in Figure S1b. The incident laser energy used was 2.41 eV. In the spectra there are two fundamental modes of graphene[2,3]: the G (~1580 cm^{-1}) and G′ (~2700 cm^{-1}) bands. On the other hand, the D band (~1360

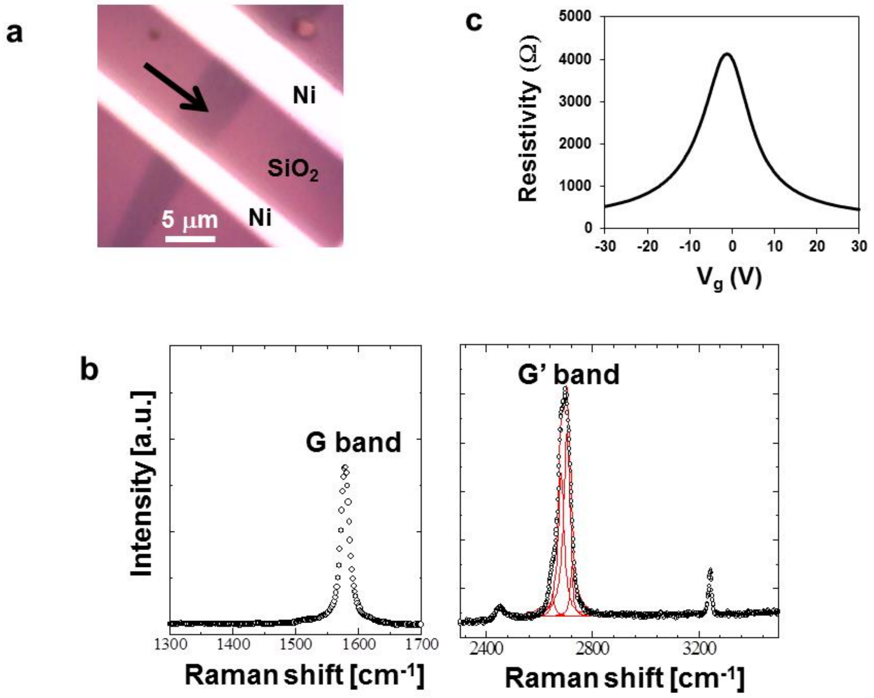

Figure S1|Characterization of the graphene used in the device in this work. (**a**) Optical micrograph of the graphene. The arrow indicates the position where the Raman spectroscopy was performed. (**b**) Raman spectra of the graphene. Black and red lines indicate decomposed peaks and a synthesized curve. (**c**) Resistance-gate voltage curve of a bilayer graphene device.

cm^{-1}), which is related to the existence of defects, cannot be seen. This indicates that graphene contains hardly any defects[2-4]. The G′ band is fitted by four peaks, which are explained within the framework of double resonant Raman scattering[2,3].

Figure S1c shows a typical resistance-gate voltage curve of a bilayer graphene transistor. The charge neutrality point (CNP) where the Fermi level is situated at the Dirac point is around V_g = 0 V, which indicates that our μ-XAS measurements are performed around CNP.

S2. Polarization dependence of μ-XAS spectra

In this work, an elliptically polarized X-ray beam was used as the incident light for microscopic x-ray absorption spectroscopy (μ-XAS) unless otherwise noted. The elliptically polarized light was used to detect the surface normal component of the XAS peaks because, in our experimental setup,

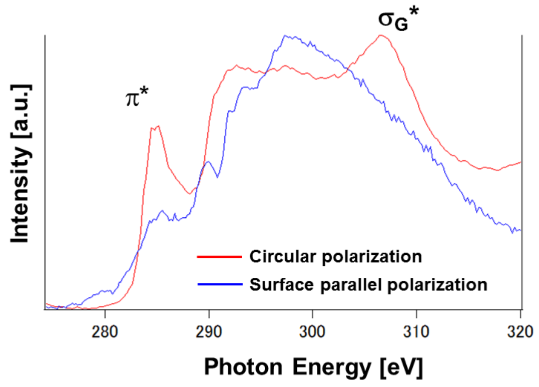

Figure S2| Polarization dependence of μ-XAS spectra. μ-XAS spectra with circularly polarized and surface parallel polarized light.

p-polarized light could not be used. The degree of the polarization is ~ 0.6[5]. The light was incident on the graphene device with a grazing angle of 74° with respect to surface normal through the PEEM installed at SPring-8[6]. Figure S2 shows the polarization dependence of the μ-XAS spectrum of the graphene. The intensity of the π* peak with surface parallel polarized light was weaker than that with the circularly polarized light. This was due to the dipole moment of the π* orbital being normal to the molecular plane. Some of the π* peak intensity remained for light polarized parallel to the surface because the polarization was incomplete.

S3. Peak decomposition of μ-XAS spectra

Figure S3 shows schematically the background correction used for the analysis of □-XAS spectra. It is assumed that the line shape of the continuum step is determined by the core hole[7]. This implies the convolution of a square step with a Lorentzian function, yielding an arctan function[7]:

$$I_{step} = H\left[\frac{1}{2} + \frac{1}{\pi}\arctan\left(\frac{E-P}{\Gamma_L}\right)\right] \qquad \text{(S1)}$$

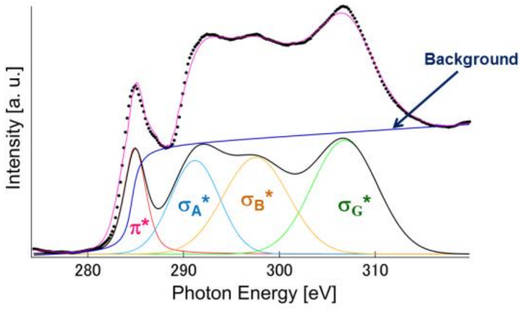

Figure S3|Peak decomposition of the μ-XAS. A typical example of peak decomposition for a μ-XAS spectrum, including the background correction in equation (S1).

where *P, H* and Γ_L are the position of the inflection, the step height of the function and the width of the step, respectively. *E* is the independent variable energy. Details are described well in the textbook by Stöhr[7]. This background correction has been applied in XAS studies of excitonic effects near the C-K edge[8], and is therefore suitable for our work.

Using this background correction, the peak decomposition of the μ-XAS spectrum was performed to extract information on the π* (~285 eV) and σ_G* (~307 eV) peaks. The decomposed σ_A* (~292 eV) and σ_B* (~297 eV) peaks, in which there are actually many peaks[9], are somewhat ambiguous with respect to shape and line width. An example is shown in Supplementary Figure S3. The fitted curve in the energy ranges of the π* and σ* peaks reproduces the experimental data.

References

1. Nagashio, K., Nishimura, T. & Toriumi, A. Contact resistivity and current flow path at metal/graphene contact. *Appl. Phys. Lett.* **97,** 143514 (2010).
2. Pimenta, M. A. *et al*. Studying disorder in graphite-based systems by Raman spectroscopy. *Phys. Chem. Chem. Phys.* **9,** 1276-1291 (2007).
3. Ferrari, A. C. *et al*. Raman spectrum of graphene and graphene layers. *Phys. Rev. Lett.* **97,** 187401 (2006).
4. Tuinstra, F. & Koenig, J. L. Raman spectrum of graphite. *J. Chem. Phys.* **53,** 1126-1130 (1970).
5. Shirasawa, K. *et al.* Development of multi-polarization-mode undulator. AIP Conf. Proc. **705**, 203-206 (2003).
6. Guo, F. Z. *et al*. Characterization of spectroscopic photoemission and low energy electron microscope using multipolarized soft X rays at BL17SU/SPring-8. *Rev. Sci. Instr.* **78,** 066107 (2007).
7. Stöhr, J. *NEXAFS Spectroscopy*. (Springer, 1992).
8. Morar, J. F. *et al.* J. L. Observation of a C-1*s* core exciton in diamond. *Phys. Rev. Lett.* **54,** 1960-1963 (1985).
9. Papagon, M. *et al*. Polarization-dependent C K near-edge X-ray absorption fine-structure of graphene. *Chem. Phys. Lett.* **475,** 269-271 (2009).
